# New Perspectives to Enhance Wastes and By-Products from Agro-Food Processing

**DOI:** 10.3390/foods12224057

**Published:** 2023-11-07

**Authors:** Maria Martuscelli, Luigi Esposito, Donatella Restuccia, Meijin Guo, Dino Mastrocola

**Affiliations:** 1 Department of Bioscience and Technology for Food, Agriculture and Environment, University of Teramo, Via R. Balzarini 1, 64100 Teramo, Italy; lesposito2@unite.it (L.E.); dmastrocola@unite.it (D.M.); 2 Department of Pharmacy, Health and Nutritional Sciences, University of Calabria, 87036 Rende, Italy; donatella.restuccia@unical.it; 3 State Key Laboratory of Bioreactor Engineering, East China University of Science and Technology, Shanghai 200237, China; guo_mj@ecust.edu.cn

The exploitation of by-products and waste from the agri-food industry represents a sustainable approach within the frame of the circular economy, the basis of the European Green Deal and ecological transition. The European Union recommends a reduction in food waste, but when this is not possible, the redirection of this waste for human consumption is recommended.

Bread and bakery products are among the most discarded food products in the world. They can be considered a high-glucose slurry; however, there is no research that considers the potential use of bread-waste hydrolysis, considering that this approach could also circumvent the costs of starch isolation, purification, and gelatinization. Sigüenza-Andrés et al. [1] studied a simultaneous hydrolysis of bread waste using α-amylase and glucoamylase; this research was carried out performing liquefaction and saccharification at the same time. This process was compared with a traditional sequential hydrolysis. The authors showed that the slurry produced with the simultaneous process had a yield higher than that obtained with sequential hydrolysis and could reduce both time and energy. 

Specifically related to the biodegradation of waste cooking oils, studies on the development of large-scale bioremediation applications were carried out. Zahri et al. [2] studied the degradation of waste canola oil and pure canola oil using a consortium of native Antarctic soil bacteria. Zahri et al. [3] also confirmed that the Antarctic soil bacterial consortium (reference BS14) are able to biodegrade canola oil. In addition, kinetic studies were carried out to examine the ability of BS14 to produce biosurfactants via a biodegradation process. Secondary mathematical equations were chosen for kinetic analyses (Monod, Haldane, etc.). Biosurfactant production was confirmed through a preliminary screening test, with further optimisation by response surface methodology (RSM). 

Recent studies reveal that by-products and waste are a significant source of bioactive compounds, which can be selectively extracted and reintroduced in the food chain to manufacture functional foods and/or be marketed as nutraceuticals. 

Innovative techniques have been tested in comparison with traditional extraction systems. Ultrasonic processing has great potential to transform waste from the food and agriculture industries into value-added products. Wu et al. [4] discussed the use of ultrasound as a green technology, comparing it to conventional chemical extraction/processing methods. They also summarized the latest developments and their future potential as energy-efficient methods to convert food and agricultural waste into value-added products. Moreover, Mrkonjić et al. [5] demonstrated that the microwave-assisted extraction (MAE) technique is an efficient approach for the extraction of biologically active compounds from food waste. In particular, these authors applied this technique to enhance *Thymus serpyllum* herbal dust, which represents a high-value source of natural antioxidants with great potential for further use in various forms within different branches of the industry. 

Aubourg et al. [6] showed the suitability of ethanol-containing green systems for the extraction of bioactive lipid compounds from squid (*Doryteuthis gahi*) by-products.

Research on food and related fields must encompass sustainability and encourage its introduction in food system production, aiming to obtain safe products with reduced preservative use. In this regard, promising results are reported by Panitsa et al. [7] regarding the use of encapsulated sodium benzoate in tubular cellulose derived from orange pulp to obtain orange juice using limited chemical preservatives. This system was capable of inhibiting the growth of spoilage microorganisms, also reducing their number (yeasts and lactic acid bacteria). 

By-products from vegetable processing are produced in large amounts each year, by both vegetable production and transformation. A critical and decisive aspect regarding the use of food by-products as food or food ingredients is their approval by the responsible government bodies. In the European Union (EU), novel foods (i.e., foods not marketed before 1997) need approval (for safety and toxicological aspects) before they can be put on the market.

As an example, the coffee chain is a large producer of interesting by-products [8]. It has been suggested to increase the income of coffee farmers by valorising coffee leaf tea consumption. Coffee leaf tea is a traditional beverage in some coffee-producing countries and was authorized in 2020 within the EU according to novel food regulations [9]. Depending on selection and processing (age of the leaves, drying, fermentation, roasting, etc.), coffee leaf tea may exhibit a wide variety of flavours. Steger et al. [10] investigated the production of coffee leaf tea (*Coffea arabica*) in El Salvador and the influence of processing steps on non-volatile compounds and volatile aroma-active compounds. 

Among other coffee by-products, coffee silver skin (CSS) is available in large amounts and is more stable due to its lower water content. Martuscelli et al. [11] carried out a study on CSS characterization and composition to verify its potentiality as a source of fibre, minerals, and bioactive molecules, while toxic minerals (e.g., nickel) were found at low levels. Martuscelli et al. [12] also investigated the use of coffee silver skin as an ingredient in meat products. In newly formulated chicken burgers, silver skin limited weight loss (after the cooking process) to 10% (1.5% addition) and 11% (3% addition), significantly lower (*p* < 0.01) than seen with the control (24%). Moreover, in cooked burgers, CSS reduced the occurrence of hexanal, and also showed good activity in the limitation of off-flavours, contrasting with the emergence of octanal, alcohols and other markers of lipid oxidation. The thiobarbituric acid reactive substances (TBARS) assay confirmed the protective effect of CSS on meat oxidation. Regarding novel food status assessment in the European Union (EU), CSS consumption is not recognized to a significant degree before 15 May 1997; therefore, consultation is currently in progress [13].

Other plant co-products such as cacao pod husks were also demonstrated to be valid new ingredients to improve the technological parameters, functional characteristics and stability of meat formulations. Delgado-Ospina et al. [14] added different cocoa pod husk flour levels as a starch replacement for reformulating frankfurters. Textural properties and sensory characteristics were affected, although these samples had higher water content, hardness, and adhesiveness, while their springiness decreased. Overall, newly formulated products demonstrated enriched dietary fibre and bioactive compounds, and had a high content of water-soluble pectins.

Bee pollen is a natural plant derivative that can be considered as a balanced, functional and health food. Only few studies exist on this natural product. Gonçalves et al. [15] expanded on the composition and phenolic profile of multifloral bee pollen from Abrantes (Portugal), as well as on its antioxidative and antidiabetic properties. These results contributed to the generation of deeper knowledge of potential health-promoting effects to develop novel pharmaceutical drugs, nutraceuticals, and dietary supplements. 

The valorisation of by-products represents a sustainable method, both from an environmental and an economic point of view. In addition to this, these matrices are rich in biologically active compounds, with positive effects on the qualitative traits of products of animal origin, as well as on animal welfare. Grass-finished beef has demonstrated wide nutritional variations, with some grass-finished beef having a considerably higher *n*-6:*n*-3 ratio compared to grain-finished beef. Krusinski et al. [16] investigated the effects of commonly used supplemental feeds on the nutritional profile of grass-finished beef. There has also been increasing interest in using grass-fed beef by-products to augment the nutrient profile of eggs within local pasture-raising systems in the United States [17]. Another important aspect is the use of waste to improve the quality of milk and derived dairy products. The introduction of grape pomace in the diet of dairy ruminants has shown several advantages, particularly related to the fatty acid profiles of milk and cheese. Bennato et al. [18] demonstrated that dietary enrichment with grape pomace did not affect the yield and chemical composition of ewe’s milk. In addition, total phenolic compounds and antioxidant activity were not affected by diet. The results of this study suggest that 10% of grape pomace can be included in the diet of lactating ewes without modifying milk gross composition but inducing significant changes in the fatty acid profile.

Finally, the prevalence of eco-friendly containers is increasing worldwide. Eco-friendly packaging materials include biodegradable and biomass products, and their continuous development is in progress. Lee et al. [19] carried out an interesting study in order to find out whether a biodegradable container made of pork gelatine can be used in industry. The containers were analysed with respect to morphological, mechanical and thermal properties, as well as biodegradability. Walnut shell powder increased the hardness, but slowed the biodegradation process. This study shows the high potentiality for applications of pork gelatine biodegradable containers for food packaging.

In conclusion, the collection of papers presented in this Editorial clearly shows that feasible and efficient solutions can be achieved by including food and agricultural wastes in a myriad of applications. These topics will be prevalent in future research globally. Finally, we cannot forget about the socio-economic benefits of the virtuous revalorisation of by-products. Due to their high quantities, they must be considered as independent production chains from which high value can be extracted.
